# Conservative Kidney Management Jumpstart: Designing a Behavioral Nudge to Jumpstart Conversations about Conservative Kidney Management between Patients and Nephrologists

**DOI:** 10.34067/KID.0000001079

**Published:** 2025-12-24

**Authors:** Susan P.Y. Wong, Olivia Gaughran, Deborah Lee, Jane Schell, Daniel Y. Lam, Grady Paden, Erin K. Kross

**Affiliations:** 1 Department of Medicine, University of Washington, Seattle, Washington; 2 Health Systems Research Center for Innovation, Veterans Affairs Puget Sound Health Care System, Seattle, Washington; 3 Department of Medicine, University of California San Francisco, San Francisco, California; 4 Department of Medicine, University of Pittsburgh, Pittsburgh, Pennsylvania; 5 Cambia Palliative Care Center of Excellence, University of Washington, Seattle, Washington

**Keywords:** geriatric nephrology, conservative management, palliative care

## Abstract

**Key Points:**

- Behavioral nudging can activate nephrologists to adopt new behaviors around discussing conservative kidney management with patients.
- Conservative kidney management Jumpstart is a nudge that offers nephrologists a framework and words-to-try to discuss health care values and conservative kidney management with patients.
- Conservative Kidney Management Jumpstart nudged nephrologists to discuss values and conservative kidney management with patients and revealed persistent challenges with these discussions.

**Background:**

Nephrologists do not routinely discuss conservative kidney management (CKM) with their patients, hindering informed decision-making about kidney failure treatments. Nudges are interventions that facilitate behavior change and may assist nephrologists with discussing CKM.

**Methods:**

We designed a behavioral nudge, called CKM Jumpstart, to assist nephrologists with discussing CKM with their patients. CKM Jumpstart was developed using human-centered design principles in 3 phases: (*1*) Discovery (March–June 2022): literature review and deliberation about the challenges to discussing CKM with an advisory panel; (*2*) Design (June–December 2022): multiple cycles of prototyping of CKM Jumpstart with input from the advisory panel and ten nephrologists across the United States; and (*3*) Implementation (April 2023–July 2025): testing a final version of CKM Jumpstart in 36 clinic visits with 19 nephrologists recruited from the greater Seattle area and conducting qualitative interviews with nephrologists about their experiences using CKM Jumpstart.

**Results:**

In the Discovery phase, we identified four major challenges to discussing CKM: (*1*) attitudes favoring dialysis as the norm; (*2*) lack of clarity about patients' health care values; (*3*) difficulty describing CKM; and, (*4*) fear of upsetting patients. The Design phase produced a prototype of CKM Jumpstart that addressed these challenges by providing a communication framework and example language that nephrologists could try to discuss patients' health care values and CKM. During the Implementation phase, all the nephrologists tried CKM Jumpstart at least once and were nudged to have conversations about values, CKM, and/or kidney failure treatment options more broadly. Nephrologists selectively used parts of CKM Jumpstart that suited their communication style. Some felt conversations occurred too early in patients' disease course and were uncertain about how to address conflicting values and treatment preferences.

**Conclusions:**

Behavioral nudging assists nephrologists with discussing health care values and CKM with patients. It can also reveal persistent challenges with having these discussions among nephrologists.

**Clinical Trial registry name and registration number:**

A Pilot Study of The CKM JumpStart Tool, NCT05753020.

## Visual Abstract

**Figure:**
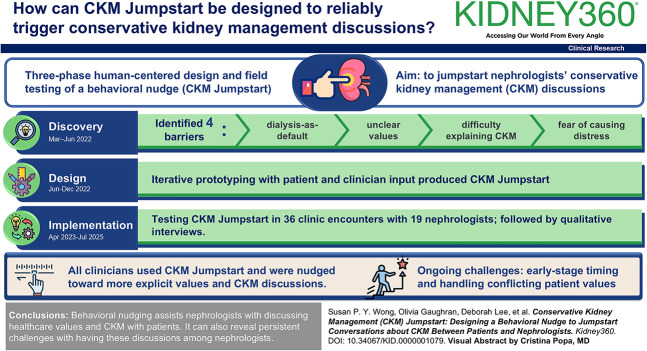

## Introduction

Conservative kidney management (CKM) is an important treatment option for patients with kidney failure who do not wish to undergo KRT or are unlikely to benefit from this treatment. The focus of CKM is the provision of kidney supportive (palliative) care to address patients' symptomatic and whole-person needs.^1^ It also includes active medical management intended to delay progression of kidney disease and manage its complications to the extent that it aligns with a patient's health care goals.

Learning about CKM supports informed decision-making and lessens ambivalence when making decisions about kidney failure treatments.^2^ However, information about CKM is very difficult for patients to access. CKM is rarely presented in kidney disease education classes,^3^ and only a few decision aids in circulation in the United States include information on CKM.^2,4^ Most nephrologists do not or are reluctant to discuss CKM,^5^ and in turn, most patients are unaware that this treatment option exists.^6,7^ Not surprisingly, most patients for whom the benefit of dialysis is least certain are being prepared for or started on this treatment.^8^

**Figure 1:**
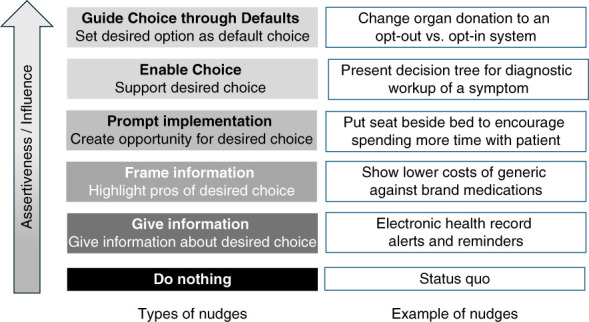
**Behavioral nudges.** Adapted from Box 3.2 from *Public health: ethical issues* from Nuffield Council on Bioethics.^27^

Behavioral nudging may be a valuable approach to activating nephrologists to discuss CKM with patients. A behavioral nudge is an intervention that influences a clinician's behavior without restricting their autonomy.^9^ Nudges can improve clinician adherence to best practices and health care delivery.^10^ Designed to be subtle and noncoercive, nudges ease clinicians into adopting a desired behavior by leveraging cognitive shortcuts (*i.e*., heuristics) that drive many everyday choices. By carefully designing how choices are presented (*i.e*., choice architecture), nudges facilitate behavior change without limiting freedom of choice, available options, or incentives. Their effect varies, with stronger effects seen from more assertive nudges (Figure 1).^11,12^ Although nudges have primarily been used to influence clinicians' treatment or diagnostic choices,^13^ there is emerging evidence that they can promote clinicians to engage in advance care planning with seriously ill patients.^14–18^

We used human-centered design principles to develop a behavioral nudge, called CKM Jumpstart, to assist nephrologists with discussing CKM with their older patients approaching kidney failure.

## Methods

Human-centered design aims to optimize fit between an intervention, its end-user, and its context.^19^ The process occurs in three phases^20^:

- Discovery: building a strong understanding of the target problem and the needs and challenges of end users.
- Design: an intervention is then iteratively designed through multiple cycles of ideation and prototyping with a small group of informants who can contribute diverse perspectives on the problem.
- Implementation: the intervention is then tested with end-users in real-world settings with attention to how it can be further refined and to generate additional insights into the problem.

The design for CKM Jumpstart was informed by an existing tool (called Jumpstart) that has been shown to be effective in clinical trials at assisting providers with initiating goals-of-care conversations with patients with serious illness.^15–17^ In brief, the core components of Jumpstart include patient-specific information obtained from either the medical record or patient surveys and a conversation framework and example language drawn from the VitalTalk communication training model (vitaltalk.org). Not only does Jumpstart provide salient information in a conspicuous fashion to enable a clinician to adopt a new behavior (*i.e*., initiating goals-of-care conversations),^21^ as part of its design, Jumpstart is given to the clinician just prior to meeting a patient, leveraging the availability heuristic in which a clinician makes decisions based on recent examples or events that are easy to recall.^22^ Jumpstart has been adapted for use with patients in inpatient and outpatient settings and with disease-specific populations, such as those with stroke, dementia, and advanced lung disease.^15–17,23–26^

The study was approved by the Institutional Review Boards at University of Washington and Veterans Affairs Puget Sound Health Care System.

### Discovery Phase

The content for CKM Jumpstart was informed by findings of literature review and evidence synthesis with an advisory panel that occurred between April and June 2022. S.P.Y.Wong (nephrologist) performed a literature review of US studies published through January 2022 that examined nephrologists' experiences with and attitudes toward discussing CKM or the option to forego dialysis with patients. We then presented the findings to an advisory panel comprising two patient advocates (D. Forfang and S. Pederson) and three physicians with expertise in nephrology (D.Y. Lam and J. Schell), palliative medicine (D.Y. Lam, G. Paden, and J. Schell), and VitalTalk methods and serious illness communication (G. Paden and J. Schell). We facilitated group discussion to identify major needs and challenges faced by nephrologists when discussing CKM. The meeting occurred over videoconference and was audio-recorded and transcribed. The meeting transcript was later shared with each panelist to allow panelists to add any further reflections.

### Design Phase

Based on the outcome of the Discovery phase, two investigators (S.P.Y.Wong and J. Randall Curtis) developed an initial prototype of CKM Jumpstart. CKM Jumpstart was designed with the goal of assisting nephrologists with discussing CKM with their patient by offering background information about their patient's health care values and a conversation framework and example language to have the conversation. Information about a patient's values would be ascertained using a validated question with patients that inquired the value they placed on extending life versus relief from pain and discomfort: “If you had to make a choice at this time, would you prefer medical care that focuses on extending life as much as possible (*i.e.,* longevity-focused care), even if it means having more pain and discomfort, or would you want medical care that focuses on relieving pain and discomfort as much as possible (*i.e.,* comfort-focused care), even if that means not living as long?”^28–30^ Patients could also respond if they were unsure about which kind of care they valued more. CKM Jumpstart was also designed to be deployed immediately before a patient's next clinic visit.

The initial prototype of CKM Jumpstart then underwent multiple rounds of review and iteration to refine its content. First, the prototype was shared with the advisory panel in the Discovery phase, who were given 2 weeks to review. The panel then convened over videoconference to discuss their feedback on the prototype. Discussions were audio-recorded and transcribed, and transcripts were shared with each panelist to solicit their additional reflections. Two investigators (S.P.Y.Wong and D. Lee) then reviewed transcripts and meeting notes and incorporated suggested changes to a prototype when recommended by at least two panelists, with J. Randall Curtis providing input to resolve any disagreement or uncertainty about revisions. The prototype underwent five rounds of review and iteration in this fashion between June and November 2022.

For feedback on layout and clarity, the prototype was then shared with ten US nephrologists with expertise in kidney palliative care and/or geriatric nephrology recruited through our professional networks. Nephrologists were provided with a paper version of the prototype and were instructed to write comments and suggested edits directly on the prototype, which was then returned to us and incorporated into the final version of CKM Jumpstart.

### Implementation Phase

We tested CKM Jumpstart in a randomized controlled pilot trial (NCT05753020) to evaluate its feasibility and acceptability. We recruited all English-speaking patients aged ≥75 years with stage 4 or 5 kidney disease receiving care at four medical centers (two academic, one federal, and one private) in the greater Seattle area and their nephrologists between April 2023 and May 2025. Patients who had made more than one visit with their current nephrologist were eligible for the study. Patients who were unable to provide informed consent or were under the care of one of the authors were excluded. As part of enrollment, patients consented to having a conversation about their health care values and CKM during their next visit with their nephrologist, although it was explained, this was not guaranteed or obligated to occur. We collected patients' demographic information (age, sex, race and ethnicity, prior education, household income, and marital status) and self-rated overall health (poor, fair, good, very good, excellent).

All patients completed the values question and half were randomly assigned to have a CKM Jumpstart generated for their nephrologists to try at their next clinic visit. A digital copy of CKM Jumpstart was emailed to the nephrologist within 4 days of the clinic visit along with an explanation of the study. A link to a brief video demonstrating how CKM Jumpstart could be used (bit.ly/CKMJumpstart) was also embedded in the email. A paper copy of CKM Jumpstart was hand-delivered to the nephrologist the day of the clinic visit by a research coordinator who briefly explained the study again and was available to answer any questions. Nephrologists were reassured that use of CKM Jumpstart was at their discretion.

To complete the Implementation phase of our human-centered design process, we conducted a nested qualitative study of the subset of nephrologists who received CKM Jumpstart to ascertain their feedback. After the clinic visit, we inquired whether nephrologists used CKM Jumpstart, recorded reasons for nonuse and invited users to provide written feedback or participate an interview. Interviews were conducted by either one of three research coordinators (with master's degrees in psychology, health education, or anthropology) trained in qualitative interviewing and who had no prior relationship to the nephrologists. Interviews took approximately 15 minutes and followed a semistructured interview guide exploring impressions of CKM Jumpstart and their conversation with the patient (Supplemental Item 2). Nephrologists could opt to have their interview audio-recorded and transcribed or have written notes taken. They received a $10 gift card for interviews. Their demographic information, including sex, years since graduating medical school, foreign medical degree status, and appointment (fellow or attending), were ascertained from medical center directories.

We performed a thematic analysis of nephrologists' feedback. Thematic analysis is a realist approach to qualitative analysis that is not bound to a specific theoretical framework, allowing flexibility in identifying patterns and themes as they are in the data.^31^ Two authors (O. Gaughran, a research coordinator with training in medical anthropology, and S.P.Y.Wong) independently reviewed each nephrologist's feedback and openly coded for themes reflecting their experiences using CKM Jumpstart. We regularly convened to assess coding consistency and completeness, confirm data saturation,^32^ discuss emergent themes, and refine theme definitions. After coding all feedback, we together reviewed all themes and illustrative quotations. Using a consensus-based approach, we assembled select themes into thematic schema reflecting how nephrologists used CKM Jumpstart and its influence on their approach to discussing CKM. We used Atlas.ti qualitative analysis software v.24 (GmbH; Berlin, Germany) to facilitate qualitative data.

## Results

### Discovery Phase

Through literature review and discussion with the advisory panel, we identified four major challenges faced by nephrologists in discussing CKM (Table 1)^5,33–43^:

- Attitudes favoring dialysis as the norm: many nephrologists perceive dialysis as the preferred treatment for patients with kidney failure and view CKM as a treatment of last resort. They do not proactively discuss CKM with patients and do not readily accept when patients express a desire not to undergo dialysis.
- Lack of clarity about patients' health care values: many nephrologists do not know their patients' health care values and whether CKM might align with these values. They do not proactively elicit these values and articulate how CKM might or might not fit with their values.
- Difficulty describing CKM: many nephrologists use language likening CKM to “end-of-life care” or “doing nothing.” They are unable to provide patients with an explanation of what CKM is, its approach to care, treatment goals and prognosis.
- Fear of upsetting patients: many nephrologists fear that discussing CKM might upset patients. They worry this conversation might be construed as “giving up” on the patient and that it could harm the patient by “taking away hope.” If patients become upset, they may lack the skills to support patients with processing this information.

**Table 1:** Major challenges with discussing conservative kidney management and how they were addressed in Conservative Kidney Management Jumpstart

Challenges Identified in Discovery Phase	Studies Reviewed in the Discovery Phase	Panelist Comments in the Discovery Phase (Panelist Role)	Elements included in the CKM Jumpstart during the Design Phase that Addressed Challenges
Attitudes favoring dialysis as the norm	Wong *et al*., 2016; Ladin *et al*., 2018; Wong *et al*., 2019a; Wong *et al*., 2019b	“It's not, is dialysis right for you? It's, you need dialysis. It's really been hard to change that culture.” (physician)	Example language validating the importance of knowing all kidney failure treatment options
Lack of clarity about patients' health care values	Schell *et al*., 2012; Combs *et al*., 2015; Grubbs *et al*., 2017; Ramer *et al*., 2018; Wong *et al*., 2019b; Baddour et al, 2019	“I met other patients who had totally different experiences than me. Talking about what's best for you. What would give you the best quality of life. What's your risk tolerance…I found out it was just the doctor diagnosed them and said, we'll get you on dialysis. Contrast to my doctor, let's talk about what would be best for you.” (patient)	Information on the patient's health care values collected through patient survey; example question to explore patient's current values; example question to explore if patient's values might change if they became ill; suggestion to revisit the patient's values in the future; suggestion to ask for help if needed to further explore values
Difficulty describing CKM	Sekkarie *et al*., 2001; Parvez *et al*., 2016; Ladin *et al*., 2018; Wong *et al*., 2019b; Cohen *et al*., 2021	“Very few providers, including nephrologists, know or feel safe in giving prognostic information. So, patients don't really get the big picture.” (physician)	Example language describing approach to care and therapeutic goals of CKM; example language of comparative survival and health care utilization between CKM and dialysis
Fear of upsetting patients	Schell *et al*., 2012; Grubbs *et al*., 2017; Ladin *et al*., 2018	“Their physicians don't even tell them, thinking, well, I don't want to scare them. There's a misconception there that patients will get fearful where really, I think those are the important times when you make life decisions.” (patient)	Information on patient's interest in learning about all kidney failure treatment options; example language normalizing conversation about CKM; example language reassuring patient that these conversations help with decision-making

CKM, conservative kidney management.

### Design Phase

The outcome of the Discovery phase (Table 1) and rounds of iteration and prototyping during the Design phase resulted in a final prototype of CKM Jumpstart (Supplemental Item 1A–C). CKM Jumpstart is a one-sheet, double-sided handout that included five main sections:

- Set-up: signposting that the patient is in the study, reassurance that the patient consented to a potential conversation about CKM if initiated by the nephrologist, and a suggestion to set aside time to have this conversation.
- Patient information on their health care values: the patient's response to the values question.
- Words-to-try: example language to explore the patient's values and perception of CKM.
- Information on CKM: example language describing the approach to care and therapeutic goals of CKM. We also provided a summary on survival, quality of life, and health care utilization associated with opting for CKM that was informed by the findings of several systematic reviews.^44–47^
- Next steps: suggestions to revisit the conversation in the future, refer to a colleague if help is needed to advance the conversation and document the conversation in the patient's medical record.

CKM Jumpstart included three versions, one for each of the three possible responses (*i.e*., longevity-focused care, comfort-focused care, unsure) a patient could give to the values question. Example language explaining how CKM might align with each of the three values differed between the versions.

### Implementation Phase

A total of 76 patients were enrolled in the larger pilot trial of which 37 were randomly assigned to have CKM Jumpstart created for their nephrologist to try at their next clinic visit. The 37 clinic visits represented 19 unique nephrologists. CKM Jumpstart could not be delivered to a nephrologist prior to one clinic visit (3%). Of the 36 opportunities to use CKM Jumpstart, nephrologists reported using it in 26 (72%), resulting in 18 nephrologists who used CKM Jumpstart at least once (Table 2). There were no differences observed in the characteristics of patients whose nephrologists used or did not use CKM Jumpstart during the visit (Table 3). Reasons for not using CKM Jumpstart included perceptions that the patient was not suitable for a conversation about CKM, it was not the appropriate time for kidney failure treatment planning, and/or there was insufficient time during the visit to use CKM Jumpstart. Nephrologists received a CKM Jumpstart an average of 2±1 times. The median time between when patients answered the values question and their subsequent clinic visit was 22 days (interquartile range, 14–38).

**Table 2:** Characteristics of nephrologists who used Conservative Kidney Management Jumpstart

Characteristics	*N*=18
Women	7 (39%)
Yr since graduating from medical school	20±11
**Medical education**	
US medical school	14 (78%)
Non-US medical school	4 (22%)
**Role**	
Nephrologist	14 (78%)
Fellow	4 (22%)

**Table 3:** Characteristics of patients for whom a Conservative Kidney Management Jumpstart tool was created

Patient Characteristics	Patients Randomized to Have a Tool Created	Patients Whose Nephrologist Used the Tool
	*N*=37	*N*=26
Women, *n* (%)	5 (14)	4 (15)
Age, mean (SD)	80 (5)	81 (5)
**Race and ethnicity, *n* (%)**		
African American or Black	3 (8)	3 (12)
Asian or Pacific Islander	2 (5)	1 (4)
Caucasian or White	26 (70)	18 (69)
Latino or Hispanic	3 (8)	2 (8)
Other^a^	3 (8)	2 (8)
**Marital status, *n* (%)**		
Married or live-in partner	20 (54)	13 (50)
Single, separated, divorced, or widowed	17 (46)	13 (50)
**Prior education, *n* (%)**		
Any high school or less	4 (11)	3 (12)
Any college or higher	33 (89)	23 (89)
**Household income, *n* (%)**		
$50,000 or less	16 (43)	11 (42)
$50,001 or more	18 (49)	13 (50)
Decline to answer	3 (8)	2 (8)
**Self-rated overall health, *n* (%)**		
Excellent	1 (3)	1 (4)
Very good	1 (3)	1 (4)
Good	16 (43)	12 (46)
Fair	11 (30)	7 (27)
Poor	8 (22)	5 (19)
**Previously spoken with nephrologist about, *n* (%)**		
Dialysis	25 (68)	17 (65)
CKM or treatment of kidney failure without starting dialysis	14 (38)	10 (39)
**Health care values reported, *n* (%)**		
Extending life	8 (22)	5 (19)
Relieving pain and discomfort	19 (51)	14 (54)
Not sure	10 (27)	7 (27)

CKM, conservative kidney management.

a Includes patients who self-reported that they did not belong to the above categories or belonged to more than one of the categories.

**Figure 2:**
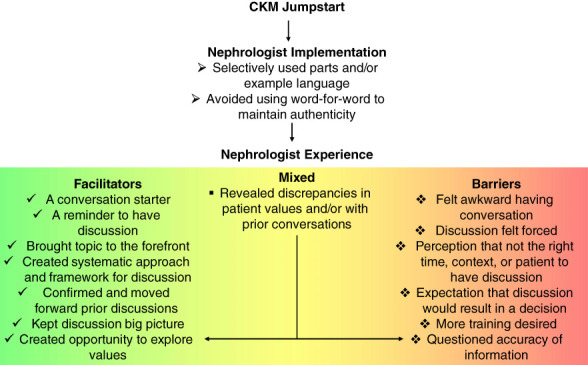
**Thematic schema reflecting nephrologists' experiences using CKM Jumpstart.** CKM, conservative kidney management.

All 18 nephrologists who used CKM Jumpstart at least once provided feedback. Figure 2 summarizes themes reflecting how nephrologists implemented CKM Jumpstart and the facilitators and barriers to having these conversations. Table 4 includes additional illustrative quotations from nephrologists. Many described CKM Jumpstart as a helpful conversation starter about patients’ values and treatment options for kidney failure. Although some felt awkward initiating conversations, they appreciated that it “brought the topic to the forefront.” Some noted the conversations felt forced earlier in the disease trajectory than preferred, as they typically waited until kidney disease was more advanced or unstable. They described difficulty spotting the right time, right context, and/or ready patient to use CKM Jumpstart. Others appreciated it as a reminder and a more systematic way to discuss patients’ values and treatment preferences.

**Table 4:** Exemplar quotations of nephrologists’ experiences using Conservative Kidney Management Jumpstart

Nephrologist ID	Quotation
**Nephrologist Implementation**	
CKMN18	I just started using the section that talked about how to bring it up, starting the conversation
CKMN29	I took a look at it before the patient came into the room. I used a few of the lead phrases and just walked through it with the patient
CKMN61	I used some of the language on there, it was a nice way to be given language of how to introduce conservative kidney therapy, and to start that conversation with a patient. I Felt like that was helpful
CKMN28	I read through it. It gave me some ideas of what to say. I said, I understand that you mentioned you were unsure, do you have any other questions, and have you been able to think about it
CKMN25	I looked at it before clinic, and last night. I used some of the phrasing in the tool to talk a little bit about CKM as an approach in and of itself as opposed to just absence of dialysis
**Nephrologist Experiences**	
Facilitators	
*CKMN31*	I like the “if your health got worse” question. I Asked him if he'd thought about if things didn't get better or got worse. I liked the open-ended nature of that question
*CKMN02*	It's very helpful because it very clearly tells you what the preference of the person is, whether they're leading toward following the conservative route or if they really want active management with dialysis or not. So that's a good screening tool, and it'll be helpful for people in busy practices
*CKMN08*	I Find most helpful as an initial prompt for the patient to think about it before coming into the beginning of this conversation. It sort of avoids that sort of out of the blue discussion if this is not something the patient has been thinking about. They have a little bit of a forewarning about an important conversation that we're about to have
*CKMN16*	It really helped me understand some of the things that he was saying. He and I have had other visits in the past, and just knowing his overall goals helped me to better kind of interpret that
*CKMN03*	I don't think I would change anything. It's always good to get another perspective, because sometimes you're surprised. Like what you think you know about a patient, and then it turns out they feel something different
Mixed	
*CKMN27*	There was a lot of words. I was almost tempted to just read it, which, that's not really what is intended. But I liked the highlighted phrases
*CKMN29*	I knew him, we've had a number of visits before. It was just completely contrary to what he had chosen previously, and the conversations that we had previously. So, either he didn't understand the question, and the tool just wasn't explicit enough for him to understand what was being asked. Or he may have been misinterpreting some of the nuances
*CKMN33*	He basically already had come to the conclusion without having the words to state it that quality mattered to him more than quantity. I was like, they thought you were unsure at the time. His granddaughter was with him and was like, I don't think he really understood what the question was because he's clearly quality focused
*CKMN18*	It went a little different than expected. So, on the tool he had indicated that he prioritized longevity over comfort. And then, he was like, I kind of think more the opposite really. But actually, it then became a really good conversation, because I said, this is what it seemed like you were implying and this is what it means to prioritize longevity over comfort. Even though it came off confusing and he seemed to contradict what he said in the tool, I think the framework of the longevity versus comfort was very helpful
*CKMN03*	We had talked about her plans for conservative care pretty much every visit and that wasn't really new. But I just mentioned the part about wanting more information, so we talked about that and it turns out that she felt adequately informed
Barriers	
*CKMN16*	I Feel like where I'm at in terms of my ability to talk to patients about this, I need some more practice. While the tool is helpful for some good wording, I need someone to observe me talking to a patient about values and goals and have them critique the way I'm doing it
*CKMN02*	This patient, her kidney function was pretty much stable for the last year. I Have not seen evidence of ongoing progression, otherwise I talk to my patients when I see evidence of progression about what's coming and what we need to discuss
*CKMN28*	I don't think it's appropriate to ask him what he values at this point. For some patients that's just that extra layer of concern that they don't need to have to think about. If I can postpone it by 10 years, that conversation, that's more important for the patient
CKMN04	I Felt unsuccessful in using the tool to get a clear decision from the patient. For me as a health care provider it is important to find out what's important to them - but I didn't feel I got an answer from this patient
*CKMN26*	Honestly, it's tough to deliver the materials in a way and timing that actually fits into clinical flow. [The conversation] cost was about 10 min of my time

All nephrologists reported selectively using parts of CKM Jumpstart, “pulling from it what seemed useful in a given conversation, as opposed to just sort of working through it.” Nephrologists did not necessarily use the example language word-for-word to maintain their authenticity. They “looked at the prompts…to help me explain the different [treatment options].” They also found “the framework of longevity versus comfort was very helpful because it kind of took a very heavy, abstract conversation and boiled it down.”

For nephrologists who had prior conversations with patients about their values and treatment preferences, the patient information provided in CKM Jumpstart and the discussion that ensued confirmed some nephrologists' prior impressions of the patient and “moved our conversation and relationship forward.” Others perceived a discrepancy between patients' responses to the values question in CKM Jumpstart and in-person discussions; a few used this as an opportunity to gain clarity on their patients' perspective, while others questioned the accuracy of the information in CKM Jumpstart. A few nephrologists expected CKM Jumpstart would result in a decision about CKM, with one saying the conversation was unsuccessful as they “did not get a clear decision from the patient.” One nephrologist added that they would appreciate more training in using CKM Jumpstart and navigating these conversations. Others appreciated how CKM Jumpstart kept the discussion big picture without expectation that patients make any decisions about kidney failure treatments.

## Discussion

We describe a systematic approach using human-centered design principles to develop a behavioral nudge that assisted nephrologists with discussing health care values and CKM with their older patients with advanced kidney disease. Our process demonstrates how a behavioral nudge can be designed to produce incremental behavior change among nephrologists supporting patient-centered decision-making about treatment of kidney failure. Our process also revealed limitations to these kinds of nudges in producing high-quality conversations about health care values and kidney failure treatment preferences.

Many interventions intended to improve patient-centered decision-making about treatment of kidney failure are costly and require significant time to train clinicians in communication skills,^43,48,49^ additional skilled staff to conduct these conversations,^50–52^ and/or separate time spent with patients outside their usual care.^53^ Consequently, these interventions can be difficult to scale up and incorporate into routine care and workflow. Although nudges are not a substitute for these more complex interventions, our findings demonstrate that they can reduce inertia in having difficult conversations and potentially set the stage for future and deeper conversations. With CKM Jumpstart, nephrologists were provided only brief orientation and no formal training in using it. To enhance its uptake into the real-world outpatient context, CKM Jumpstart was also designed to be quick and easy to read and to be provided to nephrologists in a just-in-time fashion to be used with their patients. Our findings merit exploration of whether CKM Jumpstart can be disseminated to successfully nudge behavior change around discussion of CKM among the broader nephrology community.

Nephrologists found benefit in using select parts of CKM Jumpstart, its prompts and framework, incorporating them into their existing approaches to discussing health care values and CKM with patients. The flexibility nephrologists reported in adapting CKM Jumpstart to suit their needs, patients, and clinical context highlights how well-designed nudges can support, rather than override, user agency and encourage positive behaviors. At the same time, despite all patients consenting to a values and CKM conversation, some nephrologists still felt nudged to have this conversation too early in the disease trajectory or before patients might be ready. This resonates with prior observations that decisions to forgo dialysis often occur late in illness and are precipitated by crisis.^35^ Even with CKM Jumpstart, some nephrologists could still feel awkward having these conversations and were encumbered by inconsistencies and ambiguity in patients' values and treatment preferences that CKM Jumpstart brought to light. These findings suggest that while nudges may shift behaviors, more intensive interventions are likely needed to change nephrologists' attitudes about timing of these discussions, strengthen their communication skills, and enhance their recruitment of additional help (*e.g*., specialty palliative care) to navigate complex conversations.

Our process for developing CKM Jumpstart has several notable limitations. First, CKM Jumpstart was designed for a specific population and setting. It was designed to be used with older patients and might be received differently by younger patients among whom forgoing dialysis is much less common.^8^ It was developed without input from other persons who are important to decision-making about treatment of kidney failure, such as family members, and therefore may not be suitable to support conversations involving these populations. Because study participants were predominantly White and well-educated, CKM Jumpstart might be received differently by patients from other demographic groups. CKM Jumpstart was also intended for use in clinics and by nephrologists and might not be suitable for use in other settings or by other types of health care professionals. Second, CKM Jumpstart provides only generic information on CKM. Detailed treatment plans and specific outcomes of CKM for an individual patient still require the nephrologist's input and a nuanced understanding of the patient's comorbidities, overall health, and personal needs and circumstances. Third, patients' responses to the values question were collected by research coordinators, rather than regular clinic staff. Other studies have demonstrated that collecting information about patients' care priorities can be reliably collected by clinic staff to inform care plans,^54^ supporting the potential feasibility and scalability of adopting CKM Jumpstart into routine clinical practice. Other methods to collect patients' responses to the values question, such as self-administered forms, may need to be explored to further facilitate broader implementation of CKM Jumpstart. Finally, CKM Jumpstart was designed to help nephrologists with initiating a conversation about patients' health care values and CKM and includes probes to elicit patient's values and understanding and share information. However, it alone cannot guarantee effective communication between patients and nephrologists and mutual understanding. CKM Jumpstart may also need to be coupled with other interventions to further optimize shared decision-making.

Human-centered design principles can support the development of a behavioral nudge to assist nephrologists with discussions about health care values and CKM with their older patients with advanced kidney disease as part of a routine clinic visit. Further research is forthcoming to determine its feasibility and acceptability with patients in supporting these kinds of conversations with their nephrologists and whether it will lead to greater informed and shared decision-making about CKM.

## Supplementary Material

## Data Availability

Original data generated for the study will be made available upon reasonable request to the corresponding author. Data Type: Aggregated Data; Clinical Trial Data. Reason for Restricted Access: Anonymized data for the study will be made available to interested parties upon written request and submission of a formal written research proposal that has undergone human subjects review.

## Declarative Statements

This study includes clinical experimentation and received Institutional Review Board or Ethics Committee approval. The need to obtain written informed patient consent was waived. This study includes clinical experimentation and complies with the Declaration of Helsinki. The clinical and research activities being reported are consistent with the Principles of the Declaration of Istanbul as outlined in the Declaration of Istanbul on Organ Trafficking and Transplant Tourism.

## Supplemental Material

Supplemental Item 1. (A–C) CKM Jumpstart.

Supplemental Item 2. Interview guide.
