# Supplementary material for: Conservative Kidney Management Jumpstart: Designing a Behavioral Nudge to Jumpstart Conversations about Conservative Kidney Management between Patients and Nephrologists
Source: Kidney360. 2025 Dec 24;7(5):1076–85. doi: 10.34067/KID.0000001079 (PMC13229439; doi:10.34067/KID.0000001079)
Supplement: Supplementary file 1 [file kidney360-7-1076-s001.pdf]

ASN American Society of Nephrology | JASN Journal of the American Society of Nephrology | KIDNEY360 Accessing Our World From Every Angle | CJASN Clinical Journal of the American Society of Nephrology

ASN Journal Disclosure Form

As per ASN journal policy, I have disclosed any financial relationships or commitments I have held in the past 36 months as included below. I have listed my Current Employer below to indicate there is a relationship requiring disclosure. If no relationship exists, my Current Employer is not listed.

O. Gaughran has nothing to disclose.

I understand that the information above will be published within the journal article, if accepted, and that failure to comply and/or to accurately and completely report the potential financial conflicts of interest could lead to the following: 1) Prior to publication, article rejection, or 2) Post-publication, sanctions ranging from, but not limited to, issuing a correction, reporting the inaccurate information to the authors' institution, banning authors from submitting work to ASN journals for varying lengths of time, and/or retraction of the published work.

Name: Olivia A Gaughran
Manuscript ID: K360-2025-001188R1
Manuscript Title: CKM Jumpstart: Designing a behavioral nudge to jumpstart conversations about conservative kidney management between patients and nephrologists
Date of Completion: November 3, 2025
Disclosure Updated Date: November 3, 2025

ASN American Society of Nephrology | JASN Journal of the American Society of Nephrology | KIDNEY360 Accessing Our World From Every Angle | CJASN Clinical Journal of the American Society of Nephrology

ASN Journal Disclosure Form

As per ASN journal policy, I have disclosed any financial relationships or commitments I have held in the past 36 months as included below. I have listed my Current Employer below to indicate there is a relationship requiring disclosure. If no relationship exists, my Current Employer is not listed.

E. Kross reports the following:
Employer: University of Washington; and Research Funding: National Institutes of Health.

I understand that the information above will be published within the journal article, if accepted, and that failure to comply and/or to accurately and completely report the potential financial conflicts of interest could lead to the following: 1) Prior to publication, article rejection, or 2) Post-publication, sanctions ranging from, but not limited to, issuing a correction, reporting the inaccurate information to the authors' institution, banning authors from submitting work to ASN journals for varying lengths of time, and/or retraction of the published work.

Name: Erin K. Kross
Manuscript ID: K360-2025-001188R2
Manuscript Title: CKM Jumpstart: Designing a behavioral nudge to jumpstart conversations about conservative kidney management between patients and nephrologists
Date of Completion: December 5, 2025
Disclosure Updated Date: December 5, 2025

ASN American Society of Nephrology JASN Journal of the American Society of Nephrology KIDNEY360 Accessing Our World From Every Angle CJASN Clinical Journal of the American Society of Nephrology

ASN Journal Disclosure Form

As per ASN journal policy, I have disclosed any financial relationships or commitments I have held in the past 36 months as included below. I have listed my Current Employer below to indicate there is a relationship requiring disclosure. If no relationship exists, my Current Employer is not listed.

D. Lam reports the following:
Employer: University of Washington; and Other Interests or Relationships: I am the Palliative Care Medical Advisor for Northwest Kidney Centers.

I understand that the information above will be published within the journal article, if accepted, and that failure to comply and/or to accurately and completely report the potential financial conflicts of interest could lead to the following: 1) Prior to publication, article rejection, or 2) Post-publication, sanctions ranging from, but not limited to, issuing a correction, reporting the inaccurate information to the authors' institution, banning authors from submitting work to ASN journals for varying lengths of time, and/or retraction of the published work.

Name: Daniel Y. Lam
Manuscript ID: K360-2025-001188R2
Manuscript Title: CKM Jumpstart: Designing a behavioral nudge to jumpstart conversations about conservative kidney management between patients and nephrologists
Date of Completion: December 8, 2025
Disclosure Updated Date: June 18, 2025

ASN American Society of Nephrology | JASN Journal of the American Society of Nephrology | KIDNEY360 Accessing Our World From Every Angle | CJASN Clinical Journal of the American Society of Nephrology

ASN Journal Disclosure Form

As per ASN journal policy, I have disclosed any financial relationships or commitments I have held in the past 36 months as included below. I have listed my Current Employer below to indicate there is a relationship requiring disclosure. If no relationship exists, my Current Employer is not listed.

D. Lee has nothing to disclose.

I understand that the information above will be published within the journal article, if accepted, and that failure to comply and/or to accurately and completely report the potential financial conflicts of interest could lead to the following: 1) Prior to publication, article rejection, or 2) Post-publication, sanctions ranging from, but not limited to, issuing a correction, reporting the inaccurate information to the authors' institution, banning authors from submitting work to ASN journals for varying lengths of time, and/or retraction of the published work.

Name: Deborah Lee
Manuscript ID: K360-2025-001188R2
Manuscript Title: CKM Jumpstart: Designing a behavioral nudge to jumpstart conversations about conservative kidney management between patients and nephrologists
Date of Completion: November 18, 2025
Disclosure Updated Date: November 18, 2025

ASN American Society of Nephrology | JASN Journal of the American Society of Nephrology | KIDNEY360 Accessing Our World From Every Angle | CJASN Clinical Journal of the American Society of Nephrology

ASN Journal Disclosure Form

As per ASN journal policy, I have disclosed any financial relationships or commitments I have held in the past 36 months as included below. I have listed my Current Employer below to indicate there is a relationship requiring disclosure. If no relationship exists, my Current Employer is not listed.

G. Paden has nothing to disclose.

I understand that the information above will be published within the journal article, if accepted, and that failure to comply and/or to accurately and completely report the potential financial conflicts of interest could lead to the following: 1) Prior to publication, article rejection, or 2) Post-publication, sanctions ranging from, but not limited to, issuing a correction, reporting the inaccurate information to the authors' institution, banning authors from submitting work to ASN journals for varying lengths of time, and/or retraction of the published work.

Name: Grady Paden
Manuscript ID: K360-2025-001188R2
Manuscript Title: CKM Jumpstart: Designing a behavioral nudge to jumpstart conversations about conservative kidney management between patients and nephrologists
Date of Completion: November 18, 2025
Disclosure Updated Date: November 18, 2025

ASN American Society of Nephrology · JASN Journal of the American Society of Nephrology · KIDNEY360 Accessing Our World From Every Angle · CJASN Clinical Journal of the American Society of Nephrology

ASN Journal Disclosure Form

As per ASN journal policy, I have disclosed any financial relationships or commitments I have held in the past 36 months as included below. I have listed my Current Employer below to indicate there is a relationship requiring disclosure. If no relationship exists, my Current Employer is not listed.

J. Schell reports the following:
Employer: University of Pittsburgh Medical Center; and Honoraria: uptodate.

I understand that the information above will be published within the journal article, if accepted, and that failure to comply and/or to accurately and completely report the potential financial conflicts of interest could lead to the following: 1) Prior to publication, article rejection, or 2) Post-publication, sanctions ranging from, but not limited to, issuing a correction, reporting the inaccurate information to the authors' institution, banning authors from submitting work to ASN journals for varying lengths of time, and/or retraction of the published work.

Name: Jane O. Schell
Manuscript ID: K360-2025-001188R2
Manuscript Title: CKM Jumpstart: Designing a behavioral nudge to jumpstart conversations about conservative kidney management between patients and nephrologists
Date of Completion: December 5, 2025
Disclosure Updated Date: December 5, 2025

ASN American Society of Nephrology | JASN Journal of the American Society of Nephrology | KIDNEY360 Accessing Our World From Every Angle | CJASN Clinical Journal of the American Society of Nephrology

ASN Journal Disclosure Form

As per ASN journal policy, I have disclosed any financial relationships or commitments I have held in the past 36 months as included below. I have listed my Current Employer below to indicate there is a relationship requiring disclosure. If no relationship exists, my Current Employer is not listed.

S. Wong reports the following:
Employer: University of Washington; VA Puget Sound Health Care System; and Other Interests or Relationships: Editorial Board Member of the Clinical Journal of the American Society of Nephrology.

I understand that the information above will be published within the journal article, if accepted, and that failure to comply and/or to accurately and completely report the potential financial conflicts of interest could lead to the following: 1) Prior to publication, article rejection, or 2) Post-publication, sanctions ranging from, but not limited to, issuing a correction, reporting the inaccurate information to the authors' institution, banning authors from submitting work to ASN journals for varying lengths of time, and/or retraction of the published work.

Name: Susan P.Y. Wong
Manuscript ID: K360-2025-001188R2
Manuscript Title: CKM Jumpstart: Designing a behavioral nudge to jumpstart conversations about conservative kidney management between patients and nephrologists
Date of Completion: November 17, 2025
Disclosure Updated Date: May 11, 2025
