# Supplementary material for: Conservative Kidney Management Jumpstart: Designing a Behavioral Nudge to Jumpstart Conversations about Conservative Kidney Management between Patients and Nephrologists
Source: Kidney360. 2025 Dec 24;7(5):1076–85. doi: 10.34067/KID.0000001079 (PMC13229439; doi:10.34067/KID.0000001079)
Supplement: Supplementary file 2 [file kidney360-7-1076-s002.pdf]

Supplemental Items

Table of Contents

Supplemental Item 1: CKM Jumpstart

Supplemental Item 1a: CKM Jumpstart – Longevity version

Supplemental Item 1b: CKM Jumpstart – Comfort version

Supplemental Item 1c: CKM Jumpstart – Unsure version

Supplemental Item 2: Interview Guide

JUMPSTARTING CONVERSATIONS

CONSERVATIVE KIDNEY MANAGEMENT

SET-UP:

Give yourself 5-10 mins. The conversation doesn't have to be long.

TALKING POINTS:

Your patient took a survey. Use their answers as a launching point.

Your Patient Answered	Words For You To Try
They **consented** to this conversation with you.	As part of a study, you completed a survey about what kind of care you value**. Knowing what's important to you helps with medical decision-making.** Can we go over one of your responses?"
They **value longevity** over comfort.	You noted that, if you had to choose, you would want care focused on extending life as long as possible, even if it means experiencing pain and discomfort. Can you **tell me more** about that?<br><br>**If your health got worse**, would it affect what you see as most important to you?
They **want to learn** about their options.	Few patients know about Conservative Kidney Management for kidney disease. **Can I tell you about CKM**, so that you know your options.

TURN OVER SHEET FOR WORDS ON CKM:

WORDS ON CONSERVATIVE KIDNEY MANAGEMENT

Conservative kidney management, or CKM, is a treatment pathway **focused on relieving symptoms and maintaining your independence and function without dialysis**.

We treat your kidney disease and control your symptoms with medicines and lifestyle changes. We connect you with services to help you and your family cope with your kidney disease through to the end of life.

The average patient might not live as long with CKM as they would if they had dialysis. They spend less time in the hospital, spend more time at home, and don't have to undergo any surgeries or procedures like with dialysis.

For patients with serious health problems, dialysis might not help them live longer or feel better than what can be achieved with CKM.

And so...

For patients who **value living as long as possible**, even if it means experiencing pain and discomfort, **dialysis could support this value better than CKM**.

What are your thoughts on this?

WHERE TO GO FROM HERE:

This is just a start. You can revisit their values and CKM at future visits or ask a colleague for help to take the conversation further.

WRAP-UP:

Write a short chart note. A brief summary and patient quote are enough. Your colleagues will appreciate it.

JUMPSTARTING CONVERSATIONS

CONSERVATIVE KIDNEY MANAGEMENT

SET-UP:

Give yourself 5-10 mins. The conversation doesn't have to be long.

TALKING POINTS:

Your patient took a survey. Use their answers as a launching point.

Your Patient Answered	Words For You To Try
They **consented** to this conversation with you.	As part of a study, you completed a survey about what kind of care you value**. Knowing what's important to you helps with medical decision-making.** Can we go over one of your responses?"
They **value comfort** over longevity.	You noted that, if you had to choose, you would want care focused on avoiding pain and discomfort as much as possible, even if it means not living as long. Can you **tell me more** about that?<br><br>**If your health got worse**, would it affect what you see as most important to you?
They **want to learn** about their options.	Few patients know about Conservative Kidney Management for kidney disease. **Can I tell you about CKM**, so that you know your options.

TURN OVER SHEET FOR WORDS ON CKM:

WORDS ON CONSERVATIVE KIDNEY MANAGEMENT

Conservative kidney management, or CKM, is a treatment pathway **focused on relieving symptoms and maintaining your independence and function without dialysis**.

We treat your kidney disease and control your symptoms with medicines and lifestyle changes. We connect you with services to help you and your family cope with your kidney disease through to the end of life.

The average patient might not live as long with CKM as they would if they had dialysis. They spend less time in the hospital, spend more time at home, and don't have to undergo any surgeries or procedures like with dialysis.

For patients with serious health problems, dialysis might not help them live longer or feel better than what can be achieved with CKM.

And so...

For patients who **value living as comfortably as possible**, even if it means not living as long, **CKM could support this value better than dialysis**.

What are your thoughts on this?

WHERE TO GO FROM HERE:

This is just a start. You can revisit their values and CKM at future visits or ask a colleague for help to take the conversation further.

WRAP-UP:

Write a short chart note. A brief summary and patient quote are enough. Your colleagues will appreciate it.

JUMPSTARTING CONVERSATIONS

CONSERVATIVE KIDNEY MANAGEMENT

SET-UP:

Give yourself 5-10 mins. The conversation doesn't have to be long.

TALKING POINTS:

Your patient took a survey. Use their answers as a launching point.

Your Patient Answered	Words For You To Try
They **consented** to this conversation with you.	As part of a study, you completed a survey about what kind of care you value**. Knowing what's important to you helps with medical decision-making.** Can we go over one of your responses?"
They're **unsure** whether they value longevity or comfort more.	You noted that you were unsure if you could choose between care focused on avoiding pain and discomfort as much as possible or extending life as long as possible. Can you **tell me more** about that?<br><br>**If your health got worse**, would it affect what you see as most important to you?
They **want to learn** about their options.	Few patients know about Conservative Kidney Management for kidney disease. **Can I tell you about CKM**, so that you know your options.

TURN OVER SHEET FOR WORDS ON CKM:

WORDS ON CONSERVATIVE KIDNEY MANAGEMENT

Conservative kidney management, or CKM, is a treatment pathway **focused on relieving symptoms and maintaining your independence and function without dialysis**.

We treat your kidney disease and control your symptoms with medicines and lifestyle changes. We connect you with services to help you and your family cope with your kidney disease through to the end of life.

The average patient might not live as long with CKM as they would if they had dialysis. They spend less time in the hospital, spend more time at home, and don't have to undergo any surgeries or procedures like with dialysis.

For patients with serious health problems, dialysis might not help them live longer or feel better than what can be achieved with CKM.

And so...

For patients who are **unsure** about whether they value living as long as possible or living as comfortably as possible, **CKM or dialysis is a reasonable option**.

What are your thoughts on this?

WHERE TO GO FROM HERE:

This is just a start. You can revisit their values and CKM at future visits or ask a colleague for help to take the conversation further.

WRAP-UP:

Write a short chart note. A brief summary and patient quote are enough. Your colleagues will appreciate it.

Nephrologist Interview Guide

1. How did you use the CKM Jumpstart Tool?
   a. What worked about?
   b. What didn't work about it?

2. What did your cover in your conversation?
   a. Did you talk about your patients' values? How so?
   b. Did you talk about conservative kidney management? How so?

3. How did the conversation go?
   a. What went well?
   b. What didn't go well?

4. What feedback, if any, do you have about the CKM Jumpstart Tool?

5. Anything else that you would like to share that we haven't asked you about?

Generic probes:
1. Tell me more about that.
2. Anything else?
3. Can you give me an example of [insert subject's words]?
4. What was your/their reaction?
